# Zebrafish as a tool to study schizophrenia-associated copy number variants

**DOI:** 10.1242/dmm.043877

**Published:** 2020-04-29

**Authors:** Philip D. Campbell, Michael Granato

**Affiliations:** 1 Department of Psychiatry, Perelman School of Medicine, University of Pennsylvania, Philadelphia, PA 19104, USA; 2 Department of Cell and Developmental Biology, Perelman School of Medicine, University of Pennsylvania, Philadelphia, PA 19104, USA

**Keywords:** Zebrafish, Schizophrenia, Attention-deficit/hyperactivity disorder, ADHD, Autism spectrum disorders, ASD, Copy number variant, Genetics, Psychiatry, Behavior, Intellectual disabilities, ID, Developmental delay, DD

## Abstract

Schizophrenia remains one of the most debilitating human neurodevelopmental disorders, with few effective treatments and striking consequences felt by individuals, communities and society as a whole. As such, there remains a critical need for further investigation into the mechanistic underpinnings of schizophrenia so that novel therapeutic targets can be identified. Because schizophrenia is a highly heritable disorder, genetic risk factors remain an attractive avenue for this research. Given their clear molecular genetic consequences, recurrent microdeletions and duplications, or copy number variants (CNVs), represent one of the most tractable genetic entry points to elucidating these mechanisms. To date, eight CNVs have been shown to significantly increase the risk of schizophrenia. Although rodent models of these CNVs that exhibit behavioral phenotypes have been generated, the underlying molecular mechanisms remain largely elusive. Over the past decades, the zebrafish has emerged as a powerful vertebrate model that has led to fundamental discoveries in developmental neurobiology and behavioral genetics. Here, we review the attributes that make zebrafish exceptionally well suited to investigating individual and combinatorial gene contributions to CNV-mediated brain dysfunction in schizophrenia. With highly conserved genetics and neural substrates, an ever-expanding molecular genetic and imaging toolkit, and ability to perform high-throughput and high-content genetic and pharmacologic screens, zebrafish is poised to generate deep insights into the molecular genetic mechanisms of schizophrenia-associated neurodevelopmental and behavioral deficits, and to facilitate the identification of therapeutic targets.

## Abstract (precis)

**Summary:** Specific copy number variants significantly increase the risk of schizophrenia, although their mechanisms are incompletely understood. We review the zebrafish as a tool to begin to decipher the complex biology of these rare variants.

## Introduction

Schizophrenia is characterized by perceptual and thought disturbances, disorganized behavior, emotional and social symptoms (including amotivation, asociality and apathy), and cognitive impairments (Owen et al., 2016). The clinical symptoms of schizophrenia generally manifest in adolescence or early adulthood; however, consistent with our current understanding of schizophrenia as a largely neurodevelopmental disorder, symptoms that do not meet the diagnostic criteria can often be detected earlier (Fusar-Poli, 2017). Therefore, even though schizophrenia is relatively uncommon with an estimated point and lifetime prevalence of 0.4% and 0.75%, respectively (Moreno-Küstner et al., 2018), its early and lifelong course makes it profoundly debilitating, representing one of the top 15 causes of disability worldwide (GBD 2016 Disease and Injury Incidence and Prevalence Collaborators et al., 2017). Moreover, the direct and indirect costs associated with schizophrenia are estimated to be in the hundreds of billions of dollars annually in the USA alone (Cloutier et al., 2016). To put this into perspective, although the lifetime prevalence of major depression is more than 20 times that of schizophrenia (Hasin et al., 2018), some reports have estimated that the direct and indirect costs associated with depression and schizophrenia are similar (Greenberg et al., 2015). As such, while clinicians are all too familiar with the devastating consequences schizophrenia has on individuals and families, the societal burden of schizophrenia is also significant.

Given the striking impact schizophrenia has on patients, families and society as a whole, there is great interest in the discovery and development of novel treatments. This is further motivated by the fact that the current medications approved to treat schizophrenia largely treat its ‘positive symptoms’, namely hallucinations, delusions and thought/behavioral disturbances (Miyamoto et al., 2012). There are currently no medications that effectively treat the ‘negative’ emotional and social symptoms or cognitive impairment, which are the main drivers of disability. Further, even with effective medications for positive symptoms, a large fraction, ∼30%, of patients remain treatment resistant (Elkis and Buckley, 2016), and the majority of responders experience relapse of their positive symptoms (Alvarez-Jimenez et al., 2012). Importantly, the leading cause of death among young individuals with schizophrenia is suicide, and the introduction of antipsychotics to treat young patients has had little impact on suicide rates in this population (Healy et al., 2006). As schizophrenia is a highly heritable disorder, there is hope that better understanding of its genetic architecture can provide novel insights into its pathogenesis and possible areas for intervention. Specifically, the identification of genetic risk factors or genes associated with increased risk of schizophrenia provides an entry point into investigating how these genes regulate normal brain development and how these pathways may be modulated by novel therapeutics.

## Genetics of schizophrenia

The heritability of schizophrenia is estimated to be 80-85% (Sullivan et al., 2003), and concordance rates between monozygotic twins are in the range of 41-65% (Cardno and Gottesman, 2000). Recent years have seen a vast expansion in attempts to elucidate the underlying genetics of schizophrenia. Initially, researchers had hoped to identify a handful of genetic variants (alleles) that could account for schizophrenia risk. Instead, they discovered a much more complex genetic landscape. Specifically, two main types of schizophrenia risk variants or alleles have emerged: the common, small-effect alleles and the rare, large-effect ones (Rees et al., 2015). The small-effect alleles are present relatively commonly in the general population (>1%) and, as the name suggests, impart only a small increase in the risk of schizophrenia [odds ratio (OR) 1.0-1.2]. To date, genome-wide association studies (GWAS) have identified >100 single-nucleotide polymorphism alleles (SNPs) that fall into this category (Schizophrenia Working Group of the Psychiatric Genomics Consortium, 2014; Pardiñas et al., 2018). When considered individually, these common, small-effect alleles have minimal effect on genetic risk. In contrast, when considered together through an amalgam score known as the polygenic risk score (PRS), these alleles account for significant genetic risk (OR 2.3-4.6) (Oliver et al., 2019; Zheutlin et al., 2019). Assuming that SNPs affect the function of the nearest gene, pathway-analysis approaches have implicated broad biological mechanisms that may be involved in schizophrenia pathogenesis, including synaptic signaling and roles for the immune system (Pardiñas et al., 2018). However, moving beyond broad pathobiological mechanisms and translating these schizophrenia-associated variants into cellular and molecular targets that are amenable to therapeutic intervention remains a significant challenge.

Conversely, the rare, large-effect alleles are uncommon in the general population (the most common of which, the 22q11.2 deletion, occurs in ∼0.025% of live births) but impart a substantial risk of schizophrenia, with an OR of 3 to infinity (Rees et al., 2014; Marshall et al., 2017). These ORs are similar to those of *BRCA1/2* mutations associated with breast and ovarian cancer (Kurian et al., 2017). Most large-effect alleles identified to date are copy number variations (CNVs), although some rare point mutations and small insertions/deletions (indels) have also been reported (Rees et al., 2015). CNVs are a type of structural variant that results in changes in the number of copies of a particular region of genomic DNA. These can range in size but are often quite large and span multiple genes. While risk is different across CNVs, with two large studies identifying ORs ranging from 3.39 to infinity (Rees et al., 2014; Marshall et al., 2017), it is noteworthy that all identified CNVs carry substantially more risk than the individual SNPs previously identified in GWAS (in the order of 3 to >60 times) and, depending on the CNV, confer an equal or substantially higher risk than the PRS (in the order of 1 to >20 times).

Although they are considerably less common in the population, because of their clearly defined genetic causes, large-effect alleles, particularly CNVs, provide a clearer avenue to study the cellular and molecular basis of CNV-associated schizophrenia. Further, there is some evidence to support that symptomatology (Tang et al., 2017) and response to medications (Verhoeven and Egger, 2015; Dori et al., 2017) overlap significantly between some forms of CNV-associated and idiopathic schizophrenia, suggesting that the mechanistic discoveries in CNV-associated schizophrenia might be more broadly applicable to idiopathic forms. Alternatively, it is possible that behavioral phenotypes are poor representations of the underlying pathophysiology, and that non-specific medications could benefit a broad range of patients with very different underlying pathophysiologic mechanisms. Here, mechanistic insights into particular CNV-associated schizophrenias could instead provide opportunities for personalized medicine and targeted therapeutics, which are greatly needed in the field of psychiatry. In either case, until other large-effect genetic variants are identified through whole-genome or -exome sequencing of increasingly large samples, CNVs remain the clearest genetic entry point into understanding the pathophysiologic mechanisms of schizophrenia with clear implications for patient care.

## Specific recurrent CNVs in schizophrenia

Over the past decade, research has shown that CNVs are enriched in patients with schizophrenia compared to controls (Consortium, 2008). Furthermore, studies investigating candidate loci have implicated specific recurrent CNVs, namely microdeletions and microduplications, as being more prevalent in schizophrenia patients versus controls (Rees et al., 2014). Recently, a genome-wide analysis of CNVs in 21,094 cases and 20,227 controls confirmed eight loci with recurrent CNVs with genome-wide significance that had been either previously implicated or reported as associated with schizophrenia (Table 1) (Marshall et al., 2017). These CNVs include microdeletions at 1q21.1, 2p16.3 (*NRXN1*), 3q29, 15q13.3, the distal region of 16p11.2 and 22q11.21, and microduplications at 1q21.1, 7q11.23 and at the proximal region of 16p11.2. As the genomic regions and affected genes corresponding to the risk alleles are known, these alleles provide substantial opportunity to elucidate the underlying cellular and molecular mechanisms of increased risk.

**Table 1.**
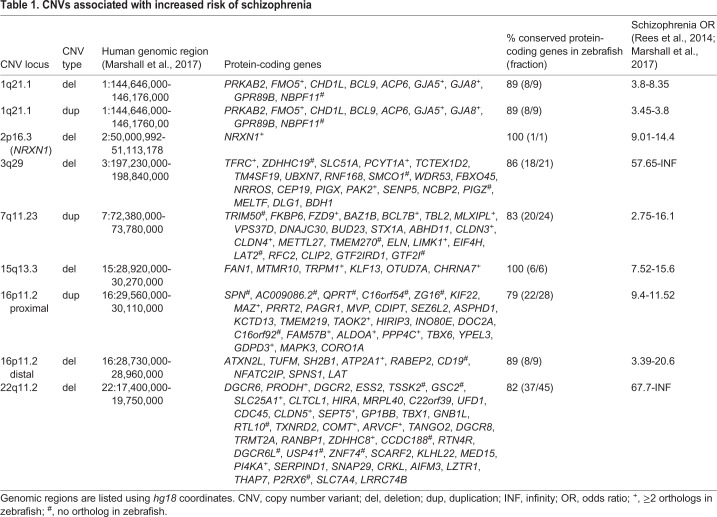
**CNVs associated with increased risk of schizophrenia**

One often-discussed potential limitation of CNV analysis is that most of the CNVs that have been identified as risk factors for schizophrenia are also risk factors for other neurodevelopmental and psychiatric disorders, in particular neurodevelopmental disorders such as autism spectrum disorders (ASD), intellectual disabilities (ID)/developmental delays (DD) and attention-deficit/hyperactivity disorder (ADHD) (Malhotra and Sebat, 2012; Chawner et al., 2019). While this is an important point to consider, it is perhaps not overly surprising as the symptom clusters that define each of these disorders overlap to a significant degree. For example, cognitive deficits are hallmarks of ASD, ID/DD and schizophrenia, social deficits are prominent features of ASD and schizophrenia, and attentional deficits span each of these diagnostic categories. Furthermore, studies have shown that there is substantial overlap between the common, small-effect variants that account for risk of many of these disorders (Anttila et al., 2018; Martin et al., 2018). In addition, a recent study has suggested that while neurodevelopmental disorder-associated CNVs predispose to many overlapping symptoms, the effects of genotype on specific phenotypes are moderate (Chawner et al., 2019). Therefore, rather than being a limitation, it appears that, in addition to providing insights into the pathogenesis of schizophrenia, systematic CNV analyses may also provide important insights into other neurodevelopmental disorders.

## CNVs: a window into schizophrenia pathogenesis

The identification of the CNVs listed in Table 1 brought substantial interest in understanding how these microdeletions and duplications lead to pathology. To date, most of this functional work has been performed in rodent models, which possess high conservation of genes and genetic synteny, allowing for modeling of specific CNVs. Mouse models for most of the deletion CNVs listed in Table 1 have been generated and shown to exhibit many phenotypes with possible relevance to schizophrenia (Etherton et al., 2009; Horev et al., 2011; Grayton et al., 2013; Fejgin et al., 2014; Portmann et al., 2014; Yang et al., 2015; Kogan et al., 2015; Tian et al., 2015; Forsingdal et al., 2016; Nielsen et al., 2017; Rutkowski et al., 2019; Stark et al., 2008; Didriksen et al., 2017; Long et al., 2006). These studies have been recently thoroughly reviewed elsewhere (Forsingdal et al., 2019). While these models are certainly useful for understanding the *en masse* changes that result from these large genomic deletions/duplications, a fine dissection and identification of the individual genes and genetic interactions that drive pathology is challenging due to inherent limitations of rodent models. Though less commonly, other model systems have also been used to investigate these CNVs and are described in Box 1.

**Box 1. Investigations into CNVs in other model systems**

While the focus of this Review is the zebrafish, other models also provide important benefits and have been used to investigate CNV pathogenesis. Non-vertebrate models, including *Drosophila* and *C. elegans*, provide many similar benefits as the zebrafish, including established genetic tools, imaging techniques and behavioral analyses. That said, fewer genes are conserved and they are conserved to a lesser degree in these animals, limiting their utility. For example, a recent review reported conservation of genes within the 22q11.2 region in *Drosophila* and *C. elegans* to be 47.8% and 37.0%, respectively, which is significantly lower compared to the over 80% conservation in both mouse and zebrafish (Guna et al., 2015). Nonetheless, owing to their suitability for high-throughput genetic manipulation and phenotypic assays, invertebrate models still provide important insights into CNV pathogenesis, with a recent study in *Drosophila* underscoring the complex genetic interactions governing the 16p11.2 region (Guna et al., 2015).

Human induced pluripotent stem cells (hiPSCs) and brain organoids are two *in vitro* models that have recently gained popularity in schizophrenia research and have been reviewed elsewhere (Balan et al., 2019). Briefly, hiPSCs are derived from patient and control individuals' somatic cells and can then be differentiated into various neurons *in vitro*, allowing for subsequent analysis of cellular phenotypes. As these cells are derived from patient samples, all genetic contributors to phenotypes are theoretically maintained and can be studied in terms of cellular processes. This is an important advantage over other models discussed herein, which generally rely on genetic engineering approaches to attempt to mimic a small number of genetic variants. Neural cells can also be cultured to create self-organizing three-dimensional structures called organoids with several neural cell types, providing a more robust *in vitro* model to study cell communication and connectivity. While powerful, these approaches also have important limitations. Specifically, schizophrenia is a behavioral and cognitive disorder, likely involving multiple cell types and developmental time points, all of which are difficult to account for in these *in vitro* models. Similarly, achieving selective synaptic connectivity and transmission that truly mimics that of an intact brain remains a significant challenge. Finally, hiPSC studies generally use few patient-derived lines, making it difficult to know how generalizable and robust results are, and the rules governing the self-organization of brain organoids are not yet fully understood, making it difficult to extrapolate findings to *in vivo* models. To date, several studies have generated hiPSCs from patients with 22q11.2 deletion with findings including disrupted microRNA processing (Zhao et al., 2015), cellular migration, neurite outgrowth, neural differentiation, neurogenic-to-gliogenic competence (Toyoshima et al., 2016) and mitochondrial dysfunction (Li et al., 2019). To our knowledge, brain organoids have not yet been used to study CNVs associated with schizophrenia.

Importantly, with one exception (*NRXN1*), all of the regions listed in Table 1 intersect multiple genes. Therefore, it is not readily apparent which genes within a defined CNV act either singly or combinatorially to give rise to specific phenotypes.

Some rodent studies have shown the importance of interactions between genes within the same CNV genomic region (Paterlini et al., 2005; Paylor et al., 2006). It is therefore surprising that systematic interrogation of all protein-coding genes within a defined CNV, including their combinatorial contributions to pathology, has not been reported, likely due to the high cost associated with rodent models. Similarly, systematic pharmacologic approaches (e.g. screens) for small molecules that may ameliorate or reverse schizophrenia-relevant phenotypes in rodent CNV models have been constrained, in part due to the challenges of performing such large-scale studies in these animals. Consequently, developing therapeutics often relies on target-based rather than on more unbiased phenotype-based approaches. Thus, a genetically tractable vertebrate model system in which to engineer allele-specific variants at high rates, combined with the ability to perform high-throughput and high-content small-molecule screens for schizophrenia-relevant phenotypes would represent a powerful tool in an expansive toolbox required to develop new therapeutics for schizophrenia. The zebrafish appears to be particularly well suited to perform such studies in a comprehensive and cost-effective manner.

## Zebrafish as a research tool in psychiatry

Alongside other commonly used animal models such as non-human primates, rodents, *Drosophila* and *Caenorhabditis* *elegans*, the zebrafish has emerged as an important model system, particularly in the fields of developmental biology and neuroscience. Like its rodent counterparts, the zebrafish genome is highly conserved with that of humans, with 82% of disease-related human genes possessing a zebrafish ortholog (Howe et al., 2013). Unlike rodent models, however, zebrafish develop *ex utero*, which facilitates analysis of the neurodevelopmental processes thought to underlie many psychiatric disorders. Furthermore, the brain develops within the first 96 h post-fertilization (hpf), and, by 120 hpf, larval zebrafish exhibit behaviors with relevance to psychiatric disorders, including sensorimotor gating (Burgess and Granato, 2007b), non-associative learning (Wolman et al., 2011) and sleep (Barlow and Rihel, 2017), which can all be assayed using automated software, allowing for high-throughput, high-content and unbiased analyses. Furthermore, the small size of larvae (several millimeters) combined with a high degree of transparency that is maintained from the embryonal through to the larval stages has allowed for brain imaging of neuronal activity during defined behaviors at single-cell and even subcellular resolutions (Marsden and Granato, 2015; Ahrens et al., 2013; Vladimirov et al., 2014; Keller and Ahrens, 2015). Recently, studies using adult zebrafish are becoming more common, as adults also possess a robust array of quantifiable behaviors with relevance to psychiatric phenotypes. Several assays that quantify fear and anxiety-like behaviors (Stewart et al., 2012), social behaviors (Geng and Peterson, 2019), sleep (Yokogawa et al., 2007), and learning and memory (Norton and Bally-Cuif, 2010) are now available, and some of these behavioral assays are coupled with live-brain imaging (Aoki et al., 2013). In addition, the currently utilized psychoactive drugs in humans have clear and predictable effects on the behavior of both zebrafish larvae and adults (Rihel et al., 2010; Wolman et al., 2011; Maximino et al., 2014), supporting the notion that, alongside genes, the neural substrates underlying psychiatric disorders also exhibit high degrees of conservation between zebrafish, mammalian models systems and humans. Further, single-cell RNA sequencing approaches have become increasingly common and have been used together with brain registration to define neuronal populations within the zebrafish brain (Pandey et al., 2018), with a recent study showing a high degree of conservation between transcriptional signatures in the habenula of mouse and zebrafish (Hashikawa et al., 2019). The zebrafish system also possesses a robust and continuously expanding toolkit for analysis of gene function. Gene knockdown and overexpression strategies are well established and easily performed (Yuan and Sun, 2009), and, with the introduction of CRISPR/Cas9-mediated genome editing, virtually any laboratory can readily generate stable zebrafish lines with gene mutations or targeted insertions (Li et al., 2016). Finally, individual matings typically result in 100-200 embryos per pair, an attribute, together with their small size, that makes zebrafish an excellent system for large-scale forward- and reverse-genetic as well as pharmacologic screens (Williams and Hong, 2016; Patton and Zon, 2001). With the numbers of progeny and tools described above, researchers can perform high-throughput and high-content screens, assaying for multiple phenotypes (Thyme et al., 2019). As a result, large-scale, unbiased, phenotype-based small-molecule behavioral screens have the potential to identify novel therapeutics that would have been unlikely to be discovered in target-based approaches in rodent systems (Williams and Hong, 2016; Hoffman et al., 2016).

As an example of the strengths of the zebrafish system and how it may be harnessed for novel discoveries in the fields of psychiatry and neuroscience, we briefly discuss a recent study by Thyme et al. (2019), which investigated common, small-effect alleles that have been associated with schizophrenia to date. Using a high-throughput CRISPR/Cas9 mutagenesis approach, the authors generated 132 zebrafish loss-of-function alleles, representing 108 previously described genomic signals (Schizophrenia Working Group of the Psychiatric Genomics Consortium, 2014). Each of the 132 mutants were then assayed for behavioral, brain activity and brain morphology phenotypes. Behavioral analyses included baseline swimming parameters, sensorimotor responses to light flash (Burgess and Granato, 2007a), dark flash (Wolman et al., 2011), acoustic stimuli (Kimmel et al., 1974) and noxious heat, and habituation (Wolman et al., 2011) and prepulse inhibition (Burgess and Granato, 2007b) to acoustic stimuli. Changes in brain volume and whole-brain activity of freely swimming 6 days post-fertilization (dpf) mutant larvae were also examined. While a small fraction displayed changes in brain morphology, over half of the mutants exhibited behavioral and/or brain activity phenotypes. Importantly, many of the targeted genes had previously not been implicated in regulating behavior and/or brain function. Moreover, for genomic regions in which the GWAS-identified small-effect variants spanned multiple genes, this study pinpointed the likely individual risk genes, as brain and behavioral phenotypes were present in mutants of one gene but not in neighboring gene mutants. Overall, this analysis prioritized 30 genes for future studies and showcased how high-throughput, unbiased assays in zebrafish can elucidate important roles in brain functioning and behavior for genes previously implicated in schizophrenia, thus identifying new potential targets for therapeutic intervention.

## Schizophrenia-associated CNVs contain highly conserved, brain-expressed genes in zebrafish

**Fig. 1.**
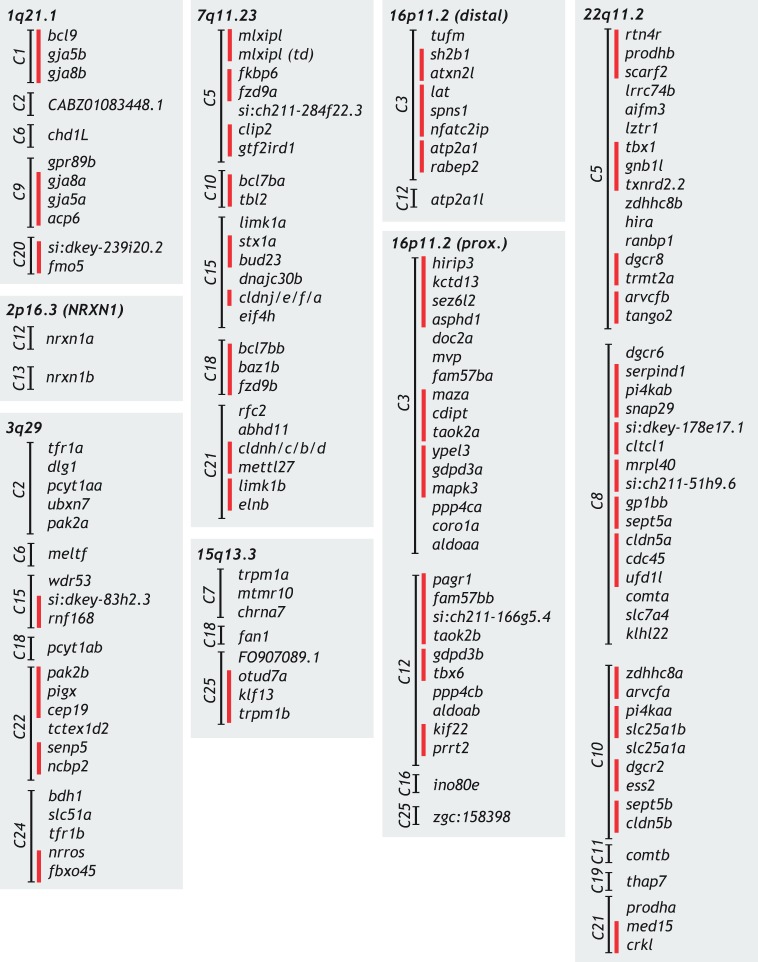
**Organization of conserved CNV genes in the zebrafish genome.** The conserved genes for each schizophrenia-associated CNV are listed and organized based on their location in the zebrafish genome. Genes are grouped by chromosome (C). Red vertical lines indicate genetic linkage, with no other annotated protein-coding genes between adjacent genes. Gene names: 1q21.1, *CABZ01083448.1*=*PRKAB2* ortholog, *si:dkey-239i20.2*=*FMO5* tandem duplication; 3q29, *si:dkey-83h2.3*=*TM4SF19* ortholog; 7q11.23, *mlxipl (td)*=*MLXIPL* tandem duplication, *si:ch211-284f22.3*=*VPS37D* ortholog; 15q13.3, *FO907089.1*=*CHRNA7* ortholog duplicate; 16p11.2 (prox.), *si:ch211-166g5.4*=*MAZ* ortholog duplicate, *zgc:158398*=*TMEM219* ortholog; 22q11.2, *si:dkey-178e17.1*=*SLC25A1* triplicate, *si:ch211-51h9.6*=*C22orf39* ortholog.

To evaluate zebrafish as a potential model to study schizophrenia-associated CNVs, we first performed literature reviews to identify the human genomic regions spanning the CNVs. We then used the Ensembl genome viewer to identify the protein-coding genes annotated within each CNV region. Finally, we identified orthologous genes in zebrafish using the ‘Orthologues view’ in Ensembl or via BLASTp searches. The results of these queries are listed in Table 1. Similar to what has been previously documented for disease-related human genes, zebrafish genes within schizophrenia-associated CNVs appear to be highly conserved, with 120/143 human genes encoding at least one ortholog in zebrafish (average 83.9%, range 79-100% for individual CNVs). Owing to the teleost-specific genome duplication (Postlethwait et al., 1998), each of the CNVs encompass at least one gene that has more than one zebrafish ortholog, with five CNVs with three or fewer genes with duplicates and three CNVs with five or more genes with duplicates. This analysis also revealed that many of the orthologous genes have been dispersed throughout the zebrafish genome, although several regions remain contiguous, suggesting possible selective pressures toward retaining linkage (Fig. 1). To further assess the utility of the model, we examined developmental expression patterns of the zebrafish orthologs using ZFIN, the Zebrafish Model Organism Database (Sprague et al., 2003). The vast majority of zebrafish orthologs with available expression data were reported to be expressed in the brain (average 86.3%, range 50-100% for individual CNVs). This is consistent with their proposed important roles in brain function and behavior, and supports the utility of the zebrafish model to study these genes.

## Zebrafish as a tool to understand CNV pathogenesis

To date, the zebrafish has been used to investigate phenotypes associated with the distal and proximal regions of 16p11.2, two CNVs associated with schizophrenia. A similar approach has also been used to study 17p13.1, a CNV associated with microcephaly and intellectual disability. While these studies illustrate many of the strengths of the zebrafish system, it is important to discuss some of their limitations prior to discussing the studies themselves. These studies largely relied on transient gene knockdown with morpholinos (MOs) and overexpression experiments to mimic gene deletion and duplication and subsequently analyzed phenotypes of interest in early embryos/larvae in an attempt to identify genes causative for specific disorder phenotypes. In recent years, MO approaches have been called into question due to off-targeting effects (Li et al., 2019) and discrepancies between mutant and morphant phenotypes (Kok et al., 2015; Stainier et al., 2017). Furthermore, ubiquitous overexpression can lead to expression in a variety of cell types and, thus, phenotypic confounds. Additionally, as injection quantities are only typically present for up to 48 h in transient MO-mediated knockdown and global mRNA overexpression assays, this precludes the analysis of more relevant behaviors exhibited only in later larval stages (5-6 dpf, see above) and in adults. Finally, as humans with schizophrenia-associated CNVs carry these genetic variants throughout their lifetime, it is perhaps more relevant to study genetic mutants wherein any genetic compensation that occurs is likely to also occur in patients with the CNV. As targeted mutagenesis and transgenesis are now readily performed with CRISPR/Cas9 (Li et al., 2016), future studies should preferentially focus on the analysis of stable mutant lines. This approach will enable analysis of gene function as it pertains to brain and behavioral phenotypes throughout the animal's lifetime. Nonetheless, to review what has been done previously and to illustrate many of the benefits of the zebrafish system in unraveling the mechanisms underlying CNV risk, we briefly discuss the results of these older studies.

## The 16p11.2 proximal deletion/duplication

In addition to increased risk of schizophrenia, the 16p11.2 proximal region is also associated with ID (Bijlsma et al., 2009), ASD (Kumar et al., 2007), seizures (Ghebranious et al., 2007) and ADHD (Lionel et al., 2011), as well as morphological abnormalities. Interestingly, CNVs in this region result in reciprocal phenotypes whereby deletion and duplication give rise to abnormalities on the opposite ends of a phenotypic spectrum. For example, 16p11.2 deletion leads to increased risk of macrocephaly and obesity, whereas 16p11.2 duplication gives rise to microcephaly and low weight (Shinawi et al., 2010; Jacquemont et al., 2011).

As an initial step towards defining the genes responsible for these phenotypes, Blaker-Lee et al. (2012) used MOs to knock down each protein-coding gene within the proximal 16p11.2 region, and then assayed embryos for gross morphological body and tail abnormalities, as well as for brain, ventricle, and eye phenotypes. Gross motor function was also assayed by analyzing the touch response. Of the 22 genes targeted, MO knockdown of 20 genes resulted in brain or eye morphological phenotypes, 16/22 resulted in body or tail phenotypes, and 14/22 displayed defective touch response, supporting the notion that this CNV contains a group of genes that is highly active in early development. To better mimic the heterozygosity present in microdeletion patients, the authors went on to analyze genes within the region that could produce phenotypes when their expression was reduced by only ∼50%. Partial knockdown of only two genes, *aldoaa* and *kif22*, fulfilled this criteria, suggesting that these genes may be particularly sensitive to the heterozygous state present in patients. As multiple genes appeared to be highly active within this region, the same group performed a follow-up gene interaction study, analyzing 162 possible pairwise combinations of 19 genes within the 16p11.2 proximal region (McCammon et al., 2017). Of the 162 gene pairs tested, 16 (10%) resulted in a ventricle phenotype and six genes appeared to be highly interactive: *fam57ba* (also known as *tlcd3ba*), *kif22*, *asphd1*, *hirip3*, *kctd13* and *sez6l2*.

In a complementary study to the initial Blaker-Lee et al. (2012) investigation, to study the reciprocal head size phenotypes in patients with this CNV, Golzio et al. (2012) overexpressed each of the 29 protein-coding genes within the 16p11.2 proximal region by injecting human transcripts into zebrafish embryos and measured head size at 4.5 dpf. Unlike in Blaker-Lee et al. (2012) and McCammon et al. (2017), overexpression of only one gene (*kctd13*) led to decreased head size. Interestingly, MO knockdown of *kctd13* increased head size, mirroring the human phenotype. However, in a separate study that highlighted the potential pitfalls of these approaches, mutant *kctd13* mice and zebrafish showed no changes in brain weight or volume (Escamilla et al., 2017).

## The 16p11.2 distal deletion/duplication

The 16p11.2 distal deletion is associated with ID, ASD and schizophrenia. Similar to the 16p11.2 proximal region, the distal region also exhibits reciprocal phenotypes: deletion is associated with macrocephaly and obesity, whereas duplication is associated with microcephaly and low weight (Bachmann-Gagescu et al., 2010; Bochukova et al., 2010). To identify the genes that contribute to the neuroanatomical defects in 16p11.2 distal CNV patients, Loviglio et al. (2017) performed overexpression and knockdown studies in zebrafish. First, each of the nine human genes within the region were overexpressed by injecting human transcripts into zebrafish embryos. Analysis of these embryos revealed that only *LAT* overexpression resulted in decreased numbers of proliferating cells in the head. Conversely, embryos injected with CRISPR/Cas9 reagents targeting endogenous *lat* developed the opposite phenotype, characterized by increased head size and total number of proliferating cells in the brain. Finally, *Lat* knockout mice also displayed enlarged total brain area, underscoring the evolutionary conservation of *LAT* gene function across species. Together, these results suggest that *LAT* may be the primary driver of the abnormal head size phenotype in patients with 16p11.2 distal deletion/duplication.

## The 17p13.1 deletion/duplication

Unlike the reciprocal phenotypes described above, a core feature of both 17p13.1 microdeletion and microduplication is microcephaly (Carvalho et al., 2014). To investigate the genes underlying this phenotype, Carvalho et al. performed overexpression and knockdown studies for all nine human genes within the smallest genomic region of overlap associated with microcephaly in patients. Overexpression of seven genes and knockdown of five of these genes resulted in a reduction of head size in zebrafish larvae. Pairwise interaction studies using subthreshold MO or mRNA levels revealed complex interactions between genes, including additive and multiplicative effects, likely reflecting an intricate crosstalk between six genes. Unlike in the 16p11.2 distal deletion/duplication, where it appears that a single gene may have a prominent role in regulating head size, for the 17p13.1 deletion/duplication it appears that multiple genes act cooperatively to do so.

## The path forward for zebrafish CNV studies

**Fig. 2.**
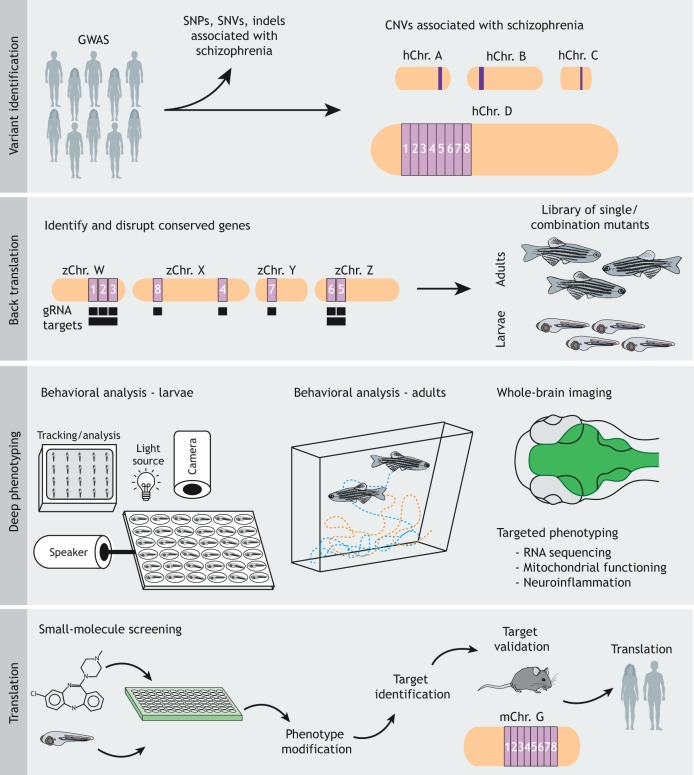
**The path forward for zebrafish CNV studies.** Clinical variants, including CNVs, are initially identified through GWAS. CNVs can be back translated into zebrafish by identifying all conserved genes (numbered 1-8 here) within a defined human CNV associated with disease (shown as hChr. D here). Researchers can use targeted mutagenesis to generate a library of single-gene as well as syntenic gene-group zebrafish mutants. A deep-phenotyping approach can be used to assess multiple behavioral phenotypes across the mutants' lifespan, combined with whole-brain imaging and more targeted phenotyping, depending on previously hypothesized mechanisms of pathogenesis. Finally, small-molecule screens for phenotype-modifying compounds can be used to identify novel targets to subsequently validate in rodent models of CNVs (shown as mChr. G here) and ultimately in humans. gRNA, guide RNA; GWAS, genome-wide association studies; hChr., human chromosome; mChr., mouse chromosome; SNP, single-nucleotide polymorphism allele; SNV, single-nucleotide variant; zChr., zebrafish chromosome.

The studies described above highlight many of the benefits of the zebrafish system as it pertains to CNV analysis (Fig. 2). In particular, identifying genes within human CNV genomic regions that are conserved in zebrafish and using gene manipulation strategies can identify the contributions of individual genes to phenotypes. These studies also illustrate the relative ease of gene interaction analysis in zebrafish, which is much more difficult to accomplish in rodent models. However, as discussed previously, the methods used in these studies have important limitations and, thus, it is worthwhile to consider how the zebrafish system might be best harnessed to investigate CNVs.

First, it is apparent that specific types of variants are much more amenable to study with zebrafish than others. For instance, duplications, which would require overexpression of multiple genes under correct spatial and temporal regulatory control, are more challenging to faithfully model than deletions, which require relatively straightforward mutagenesis approaches. Second, as patients with these deletion CNVs are hemizygous and risk is thought to result from loss of the genetic material contained within the region, it is important to consider whether heterozygous or homozygous loss-of-function mutants should be assayed. In mouse, there are examples of genes within the 22q11.2 region that result in behavioral phenotypes when in the heterozygous state (Paylor et al., 2006; Stark et al., 2008); however, others require homozygosity to express phenotypes (Gogos et al., 1999; Mukai et al., 2004; Suzuki et al., 2009). One possible explanation for this is that multiple genes within the regions interact and, thus, in some cases, loss of a single copy of a gene is not sufficient if the other genes within the region are present with both copies. As such, studying heterozygotes, homozygotes and combinatorial transheterozygotes seems the optimal choice. Third, as mentioned previously, several schizophrenia-associated CNVs are also associated with other neurodevelopmental disorders and more widespread brain abnormalities. In these contexts, it is reasonable to question whether it is useful to study behavioral phenotypes that could be due to a multitude of contributing factors. Perhaps most informative and where zebrafish might be most useful would be in identifying specific genes or gene combinations that lead to reproducible behavioral phenotypes without affecting gross brain structure. Such mutants would also serve as excellent tools for the identification of novel small-molecule modulators of these behavioral phenotypes, which could then be further tested in mammalian models. Finally, while these recurrent CNVs substantially increase the risk of schizophrenia, individual CNVs are not sufficient to cause the disorder. As such, there must be other, likely environmental and genetic, factors at play. Zebrafish models could be used to assess the underlying mechanisms of this variability. For instance, comparing RNA sequencing results between groups of individuals with varying phenotypes could be used to assess genetic compensation, and exposing animals to stressors and assessing phenotype variability could be used to understand how environmental conditions protect against or aggravate the phenotypes of interest.

With this in mind, we propose a path forward for future zebrafish CNV studies. Using genetic mutants, an initial analysis of CNV regions could start with screening conserved genes for behavioral phenotypes. Furthermore, including heterozygous mutants that faithfully recapitulate the heterozygosity present in microdeletion patients should facilitate the interpretation of experiments to evaluate combinatorial (behavioral) phenotypes. Should heterozygotes not develop phenotypes, homozygotes should also be analyzed. Moreover, many genes within CNV regions of the zebrafish genome have retained some degree of collinearity with the human counterparts (Fig. 1). This provides a unique opportunity to assess true genetic interactions involving multiple genes by generating deletions of genomic regions encompassing multiple genes, an approach now available in zebrafish (Xiao and Zhang, 2016). Collections of mutant zebrafish lines of all genes within CNV regions will then be extremely useful for two approaches with clear translational emphasis. First, using the tools described in previous sections, these mutants will provide opportunities to further dissect the cellular and molecular mechanisms underlying the behavioral phenotypes associated with genes contained within CNV regions. Second, identification of behavioral phenotypes in strains with mutations in individual or combinations of genes will provide a platform for unbiased phenotype-based pharmacologic screens to identify novel modulators of abnormal brain outputs. The compounds identified in such screens can also be assayed for their ability to modulate behaviors in adult zebrafish to assess differential lifetime effects. And, as many neural substrates appear to be conserved between humans and zebrafish (Wolman et al., 2011; Maximino et al., 2014; Bruni et al., 2016), small molecules found to modulate behaviors in zebrafish have the potential to also modulate behaviors in mammals, including humans. As such, these two complementary approaches will help (1) to identify the molecular and cellular targets with potential relevance to pathogenesis, and (2) to identify novel modulators of abnormal behavioral outputs with potential applicability to patients.

## Conclusion

Despite intense efforts to decipher the molecular genetic mechanisms of and to develop effective therapeutics for this devastating disorder, schizophrenia remains highly debilitating. Over the past two decades, researchers have identified specific CNVs associated with increased risk of schizophrenia, and analysis of the pathogenesis of these defined genetic lesions appears to be one of the best entry points into the underpinnings of this disorder. Using rodent modeling of these genetic lesions for the identification of new therapeutic options for patients has proven extremely difficult. This is in part due to the prohibitively high costs associated with generating and analyzing comprehensive mutant allele combinations contained within individual CNV regions, and the limited throughput of drug testing in rodent models. Given these difficulties and limitations, zebrafish provide a powerful addition to the investigative toolbox, given its versatile suite of high-throughput molecular tools to engineer risk alleles for CNV regions that contain large numbers of genes, individually or in almost endless combinations. This, paired with validated high-throughput assays that measure schizophrenia-relevant animal behaviors, platforms for high-throughput and high-content small-molecule screening will enable powerful dissection of genetic risk alleles and facilitate the identification of novel therapeutics that modulate relevant behavioral phenotypes. As such, the zebrafish model holds the promise of high-impact and translational discoveries with relevance not only to CNV-specific phenotypes, but, perhaps, to schizophrenia more broadly.
